# Selective mass enhancement close to the quantum critical point in BaFe_2_(As_1−*x*_P_*x*_)_2_

**DOI:** 10.1038/s41598-017-04724-3

**Published:** 2017-07-04

**Authors:** V. Grinenko, K. Iida, F. Kurth, D. V. Efremov, S.-L. Drechsler, I. Cherniavskii, I. Morozov, J. Hänisch, T. Förster, C. Tarantini, J. Jaroszynski, B. Maiorov, M. Jaime, A. Yamamoto, I. Nakamura, R. Fujimoto, T. Hatano, H. Ikuta, R. Hühne

**Affiliations:** 1 Institute for Solid State Physics, TU Dresden, 01069 Dresden, Germany; 2 IFW Dresden, Helmholtzstrasse 20, 01069 Dresden, Germany; 3 Department of Crystalline Materials Science, Graduate School of Engineering, Nagoya University, Furo-cho, Chikusa-ku, Nagoya 464-8603 Japan; 4 Lomonosov Moscow State University, GSP-1, Leninskie Gory, Moscow, 119991 Russian Federation; 5 Karlsruhe Institute of Technology, Institute for Technical Physics, Hermann-von-Helmholtz-Platz 1, 76344 Eggenstein-Leopoldshafen, Germany; 6 Hochfeld-Magnetlabor Dresden (HLD-EMFL), Helmholtz-Zentrum Dresden-Rossendorf, 01314 Dresden, Germany; 7 NHMFL, Florida State University, Tallahassee, FL 32310 USA; 8 MPA-CMMS, Los Alamos National Laboratory, Los Alamos, NM 87545 USA; 9 Department of Applied Physics, Tokyo University of Agriculture and Technology, 2-24-16 Nakacho, Koganei, Tokyo 184-8588 Japan

## Abstract

A quantum critical point (QCP) is currently being conjectured for the BaFe_2_(As_1−*x*_P_*x*_)_2_ system at the critical value *x*_*c*_ ≈ 0.3. In the proximity of a QCP, all thermodynamic and transport properties are expected to scale with a single characteristic energy, given by the quantum fluctuations. Such a universal behavior has not, however, been found in the superconducting upper critical field *H*_c2_. Here we report *H*_c2_ data for epitaxial thin films extracted from the electrical resistance measured in very high magnetic fields up to 67 Tesla. Using a multi-band analysis we find that *H*_c2_ is sensitive to the QCP, implying a significant charge carrier effective mass enhancement at the doping-induced QCP that is essentially band-dependent. Our results point to two qualitatively different groups of electrons in BaFe_2_(As_1−*x*_P_*x*_)_2_. The first one (possibly associated to hot spots or whole Fermi sheets) has a strong mass enhancement at the QCP, and the second one is insensitive to the QCP. The observed duality could also be present in many other quantum critical systems.

## Introduction

In most of unconventional superconductors, a quantum critical point (QCP) of charge or spin density wave (CDW/SDW) states lies beneath the superconducting dome^1–4^. Low-energy quantum fluctuations in the vicinity of a QCP lead to non-Femi liquid (nFL) behavior in the normal state and a strong enhancement of the effective electron mass (m*). A good example is given by heavy fermion superconductors. In some of these systems the maximum superconducting transition temperature (*T*_c_) coincides with the position of the expected QCP of the magnetic phase^4^. The presence of a QCP beneath the superconducting dome is evidenced by a strong enhancement of the superconducting specific heat jump Δ*C*/*T*_c_ at *T*_c_ and the slope of the upper critical field $|{H}_{{\rm{c}}2}^{^{\prime} }|=|{\rm{d}}{H}_{{\rm{c}}2}/{\rm{d}}T|$ normalized by the critical temperature in the vicinity of *T*_c_^5^.

In multi-band iron-based superconductors (FBS), the maximum of *T*_c_ is usually linked to the expected position of a QCP of the SDW phase^6^. Evidence for a zero-temperature second order magnetic transition with pronounced quantum fluctuations was found for optimally doped BaFe_2_(As_1−*x*_P_*x*_)_2_ by various measurements in the normal state^7–12^. Therefore, it is considered to be a classical example of unconventional superconductivity emerging in the vicinity of a magnetic state^13, 14^. However, no doping dependence of the scattering rates expected for a QCP scenario was observed in recent angle-resolved photoemission spectroscopy (ARPES) studies^15^. In the superconducting state, a divergent quasiparticle effective mass (m*) above the QCP of the SDW phase was suggested based on specific heat^16^ and penetration depth measurements^17, 18^ as well as predicted by theoretical studies^19, 20^. However, *H*_c2_ at low *T* and its slope near *T*_c_ are insensitive to the QCP^21^. This behavior is seemingly in contradiction to many other experimental observations. To resolve this puzzle we investigated in detail the temperature dependence of *H*_c2_ for BaFe_2_(As_1−*x*_P_*x*_)_2_ single-crystalline thin films in a wide range of P-doping. The obtained data can be described in an effective two-band model with qualitatively different doping dependences of the Fermi velocities (*v*_F_). Namely, *v*_F1_ is indeed nearly featureless across the QCP implying a doping independent ${m}_{1}^{\ast }$. On the other hand, *v*_F2_ is strongly doping-dependent, in accord with the almost divergent logarithmic enhancement of ${m}_{2}^{\ast }$ observed in many other experiments.

## Results

### Electronic phase diagram of BaFe_2_(As_1−*x*_P_*x*_)_2_

**Figure 1:**
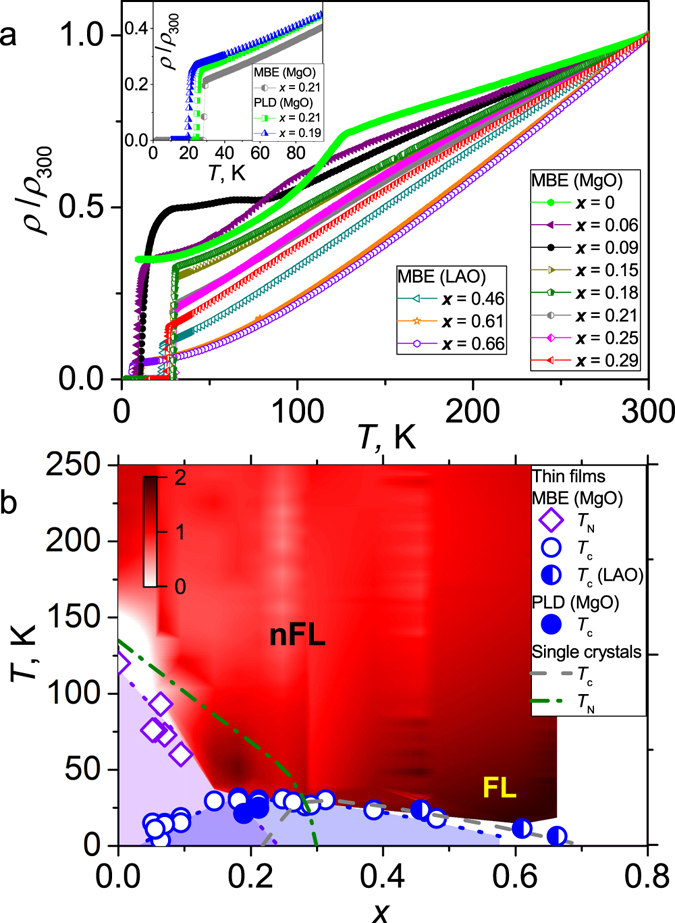
(**a**) The temperature dependence of the normalized resistivity *ρ*/*ρ*_300K_ of BaFe_2_(As_1−*x*_P_*x*_)_2_ films prepared by MBE. Closed symbols - underdoped, half closed symbols - optimally doped, and open symbols - overdoped samples. Inset: The normalized resistivity traces for BaFe_2_(As_1−*x*_P_*x*_)_2_ thin films with the similar P-doping prepared by PLD and MBE. (**b**) The phase diagram of BaFe_2_(As_1−*x*_P_*x*_)_2_ thin films (symbols). The data of BaFe_2_(As_1−*x*_P_*x*_)_2_ single crystals (dashed lines)^14, 25^ are also shown for comparison. The whole phase diagram for the thin films prepared on MgO substrates is shifted to lower doping levels compared to that of the single crystals and films prepared on LAO substrates. The shift of the phase diagram is substrate-dependent due to different in-plane strains. The contour plot of the doping and temperature dependence of the exponent *n* is obtained from the data shown in Fig. 1a and in ref. 23 assuming *ρ* = *ρ*_0_ + *AT* ^*n*^. The position of the QCP is around *x*_c_ ~ 0.25 for the films prepared on MgO substrates. The positions of the QCP for the single crystals and films prepared on LAO substrates do nearly coincide at *x*_c_ ~ 0.30. For further details see text. The doted lines are guides to the eyes.

BaFe_2_(As_1−*x*_P_*x*_)_2_ epitaxial thin films were grown by molecular beam epitaxy (MBE)^22, 23^. The investigated MBE thin films have high crystalline quality with *T*_c_ values above 30 K at optimal doping level. Some of the films were prepared by pulsed laser deposition (PLD). The PLD films have slightly reduced *T*_c_ at similar doping levels compared to the films prepared by MBE as shown in inset of Fig. 1a. This result is consistent with previous studies^24^. To construct the phase diagram of our thin films, we analyzed the temperature dependence of the resistivity for various doping levels shown in Fig. 1a. The phase diagrams of the BaFe_2_(As_1−*x*_P_*x*_)_2_ thin films and single crystals^14, 25^ are shown in Fig. 1b. The whole phase diagram for the thin films prepared on MgO substrates is shifted to lower doping levels compared to that of the single crystals. The shift of the phase diagram, as it was shown in previous studies, is substrate-dependent due to different in-plane strain^22, 23, 26–28^. In particular, the in-plane tensile strain for the films grown on MgO modifies slightly the position of the bands resulting in the observed difference between the phase diagrams of thin films and single crystals^28^. On the other hand, the amount of strain for the films grown on LaAlO_3_ (LAO) is negligibly small resulting in the same phase diagram as for single crystals^23^.

We assumed that the temperature dependences of the resistivity (Fig. 1a) can be described by *ρ* = *ρ*_0_ + *AT* ^*n*^ in the normal state above the superconducting and magnetic transition temperatures. This general expression has been frequently employed in the quantum critical region, where *n* = 1 at the QCP and *n* = 2 in a Fermi liquid (FL) state^8, 14^. The contour plot in Fig. 1b illustrates the temperature and doping dependences of the exponent $n=\frac{T{d}^{2}\rho /d{T}^{2}}{d\rho /dT}+1$, as calculated using experimental temperature dependences of the resistivity. In this analysis we exclude the data close to the SDW transition, where $d\rho /dT\mathop{ < }\limits_{ \tilde {}}0$ (white region in Fig. 1b). The region in the phase diagram with nFL behavior is similar to the single crystals: the exponent *n* shows a V-shape; however, it shifts to lower doping level. This allows us to estimate the critical doping level for thin films on MgO substrates as ${x}_{{\rm{c}}}\approx 0.25\pm 0.03$, which is slightly lower than ${x}_{{\rm{c}}}\approx 0.3$ reported for single crystals^14^. For the films prepared on LAO substrate we assumed that the position of the QCP coincides with the one for the single crystals due to the close similarity between their phase diagrams as discussed above.

### Upper critical field

**Figure 2:**
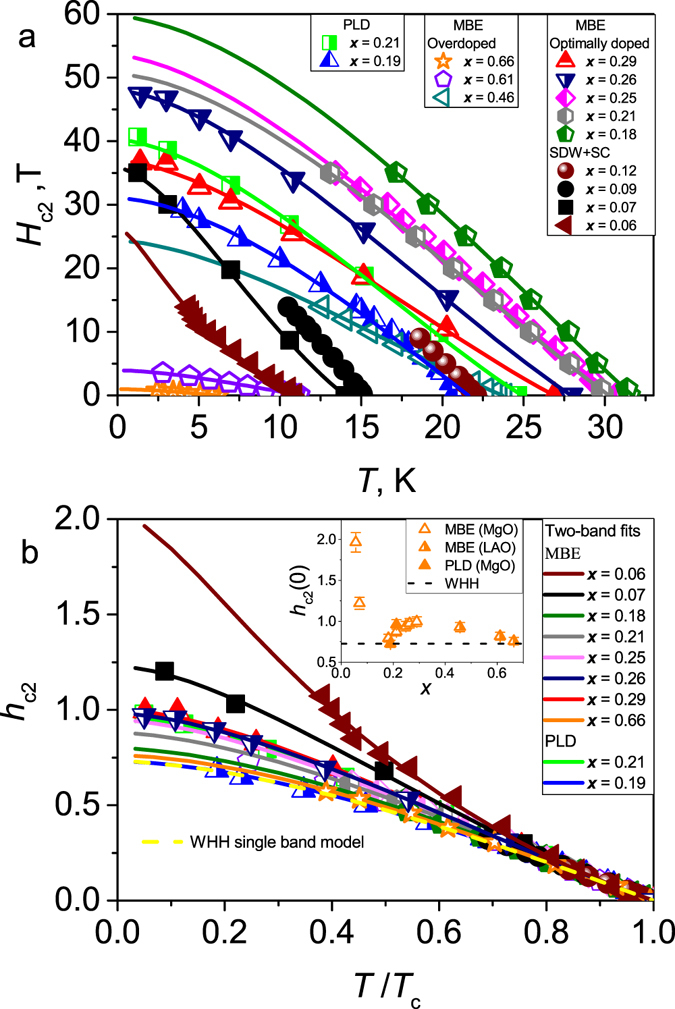
(**a**) Temperature dependences of the upper critical field *H*_c2_ of BaFe_2_(As_1−*x*_P_*x*_)_2_ thin films with various doping levels for the magnetic field applied along the *c*-axis. Closed symbols - underdoped, half-open symbols - optimally doped, and open symbols - overdoped samples, solid lines - two-band fits. (**b**) The reduced field ${h}_{{\rm{c2}}}=\frac{{H}_{{\rm{c}}2}}{-{H}_{{\rm{c}}2}^{^{\prime} }{T}_{{\rm{c}}}}$ as a function of *T*/*T*_c_ for the data shown in Fig. 2a, solid lines - two-band fits (the same as in Fig. 2a), dashed line - single-band WHH model. The inset shows the doping dependence of the *h*_c2_ values extrapolated to *T* = 0 K. The deviation of the experimental data from the single-band curve indicates a relevance of multi-band effects for the temperature dependencies of *H*_c2_. This deviation is doping - dependent as shown in the inset and can be described by a two-band model for a clean superconductor with dominant interband couplings. For further details see text.

The temperature dependences of *H*_c2_ for BaFe_2_(As_1−*x*_P_*x*_)_2_ thin films with various doping levels for fields parallel to the *c*-axis are shown in Fig. 2. The temperature dependence of *H*_c2_ is strongly affected by the amount of doping. To compare the data of samples with different doping levels, we plot the reduced field ${h}_{{\rm{c2}}}=\frac{{H}_{{\rm{c2}}}}{-{H}_{{\rm{c2}}}^{^{\prime} }{T}_{{\rm{c}}}}$ versus the reduced temperature *t* = *T*/*T*_c_ in Fig. 2b, where ${H}_{{\rm{c2}}}^{^{\prime} }$ is the extrapolated slope of *H*_c2_ at *T*_c_. For the strongly overdoped, and slightly underdoped samples, 0.15 < *x* < 0.21, the experimental *h*_c2_ data are close to the prediction of the single-band Werthamer-Helfand-Hohenberg (WHH) model which includes only the orbital pair-breaking effect^29^. For other doping levels, the experimental *h*_c2_ data deviate from the single band fit. The doping dependence of *h*_c2_(0) extrapolated to zero temperature is shown in the inset of Fig. 2b. The *h*_c2_(0) values exhibit a broad maximum around optimal doping *x*_c_. Additionally, *h*_c2_(0) is strongly enhanced in the coexistence state between SC and magnetism, where the SDW transition temperature *T*_N_ > *T*_c_.

The doping evolution of the temperature dependences of *H*_c2_ can be described by the two-band model for a clean superconductor as proposed by Gurevich^30, 31^ assuming dominant interband coupling, ${\lambda }_{12}{\lambda }_{21}\gg {\lambda }_{11}{\lambda }_{22}$, as expected for *s*_±_ superconductors. The expression for $B\parallel c$ is given in the Supplementary material Eq. S1. A small value of the intraband coupling constants *λ*_11_ = *λ*_22_ ~ 0.1 affects the resulting Fermi velocities within 10%, only around optimal doping (see Fig. S7) and has a negligible effect for overdoped samples. Therefore, to reduce the number of fitting parameters, we adopted zero intraband pairing constants *λ*_11_ = *λ*_22_ = 0. In this case, the superconducting transition temperature is related to the coupling constants by ${T}_{{\rm{c}}}=1.14\,{{\rm{\Omega }}}_{{\rm{sf}}}{{\rm{e}}}^{(-1/{\lambda }_{12}{\lambda }_{21})}$. We considered two different values of the characteristic spin fluctuation energy Ω_sf_: 100 K and 62 K, in order to take into account  the observed softening of the spin fluctuations spectrum at the QCP^32^. We assumed also that the paramagnetic pair breaking is negligibly weak, ${\alpha }_{{\rm{M}}}\ll 1$, as suggested by the small electronic susceptibility of BaFe_2_(As_1−*x*_P_*x*_)_2_, where the Maki parameter ${\alpha }_{{\rm{M}}}={2}^{\mathrm{1/2}}{H}_{{\rm{c2}}}^{{\rm{orb}}}/{H}_{{\rm{p}}}$, defined by the ratio between the orbital critical field ${H}_{{\rm{c2}}}^{{\rm{orb}}}$ and the Pauli limiting field *H*_p_, quantifies the strength of the paramagnetic pair breaking (see also the Supplementary material). This assumption is consistent with a relatively small Knight shift of BaFe_2_(As_1−*x*_P_*x*_)_2_^12^. The result of the fit is shown in Fig. 2, and the obtained fitting parameters are given in the Supplementary Tables (Tables S1 and S2).

## Discussion

**Figure 3:**
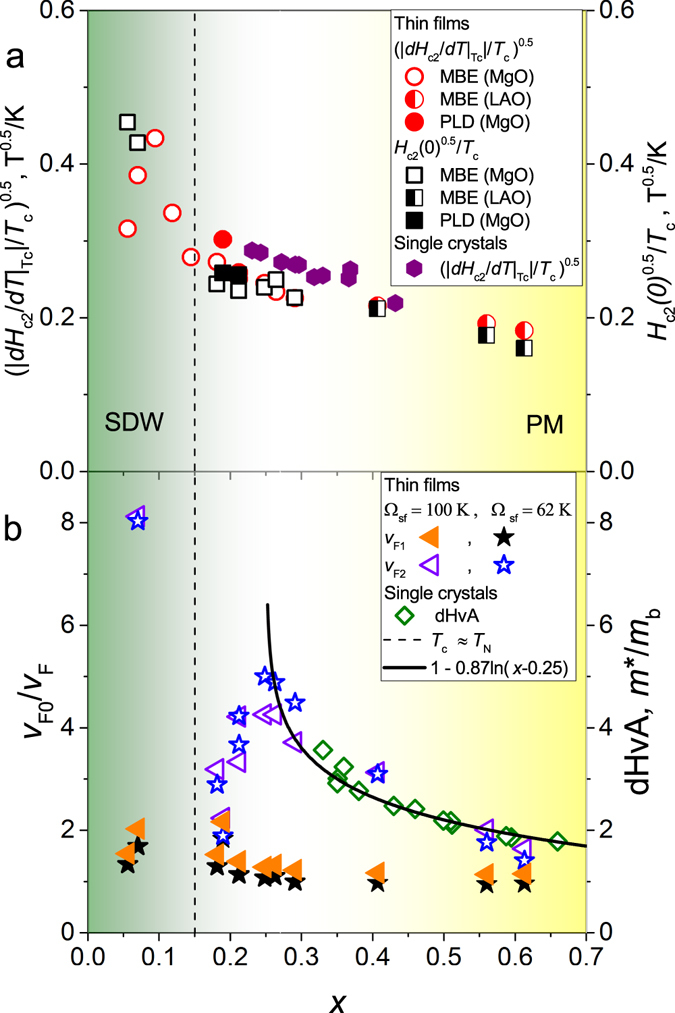
((**a**) left axis) The normalized slope of the upper critical field ${(|{H}_{{\rm{c}}2}^{^{\prime} }|/{T}_{{\rm{c}}})}^{0.5}$ at *T*_c_ and ((**a**) right axis) the normalized upper critical field (*H*_c2_)^0.5^/*T*_c_ extrapolated to *T* = 0 K using the fits shown in Fig. 2 versus the P-doping level *x*. Both these quantities are related to the charge carrier effective mass m* as discussed in the text. The single - crystal data are taken from ref. 21. ((**b**) Left axis) The inversed normalized Fermi velocities *v*_F0_/*v*_F1_ and *v*_F0_/*v*_F2_ in a two-band model are obtained from the fits shown in Fig. 2. ((**b**) Right axis) The normalized effective quasiparticle mass m*/*m*_b_ obtained from the dHvA data^7, 16^. The *v*_F0_ values are chosen to fit dHvA data, *v*_F0_ = 1.3 · 10^7^ and 1.1 · 10^7^ cm s^−1^ for Ω_sf_ = 100 K and 62 K, respectively. All the data of the thin films grown on LAO, and single- crystal data are shifted by Δ*x* = −0.05 to meet the QCP of the thin films grown on MgO according to Fig. 1b. The solid line is a fit to a phenomenological divergence of the effective mass near a QCP $1/{v}_{{\rm{F1}}}\propto {m}^{\ast }\propto 1+0.87\,\mathrm{ln}(x-{x}_{{\rm{c}}})$, refs 8 and 13, with *x*_c_ = 0.25. The dashed line denotes approximately the P doping value where *T*_c_ = *T*_N_.

The doping dependencies of ${|{H}_{{\rm{c2}}}^{^{\prime} }/{T}_{{\rm{c}}}|}^{0.5}$ extrapolated to *T*_c_, and the ${H}_{{\rm{c}}2}^{0.5}/{T}_{{\rm{c}}}$ extrapolated to *T* = 0 K are shown in Fig. 3a. According to the BCS theory for clean superconductors, these values are proportional to the quasiparticle effective mass (m*). As it was pointed out in ref. 21, ${|{H}_{{\rm{c}}2}^{^{\prime} }/{T}_{{\rm{c}}}|}^{0.5}$ should have a peak-like maximum at the QCP of the SDW phase since m* is strongly enhanced near optimal doping on the whole Fermi surface according to various experimental data^7, 16, 17^. However, this is not the case: ${|{H}_{{\rm{c}}2}^{^{\prime} }/{T}_{{\rm{c}}}|}^{0.5}$ and ${H}_{{\rm{c2}}}{\mathrm{(0)}}^{0.5}/{T}_{{\rm{c}}}$ are nearly featureless at optimal doping (*x*_c_ ~ 0.25) in accord with ref. 21. Both the single crystals and our MBE films have high *T*_c_ values of about 30 K at optimal doping indicating similar low impurity scattering rates. The slightly higher ${|{H}_{{\rm{c}}2}^{^{\prime} }/{T}_{{\rm{c}}}|}^{0.5}$ values of the single crystals compared to those of the MBE films are probably related to the different experimental methods used for the evaluation of *H*_c2_. Also, *H*_c2_ of the PLD films follows the same trend in spite of a lower *T*_c_ and residual resistivity ratio (inset of Fig. 1a) as compared to the MBE films. Therefore, we believe that the observed doping dependence of *H*_c2_ is not affected essentially by impurity scattering rates and related instead mainly to the changes of *v*_F_ and the coupling constants.

*H*_c2_ of multi-band unconventional *s*-wave superconductors with dominant interband coupling is limited by the largest *v*_F_ in the usually considered pronounced *s*_±_-regime^30, 31^. Therefore, in the case of a strong *v*_F_ asymmetry between different bands, the larger Fermi velocity (*v*_F1_ in our notation) dominates *H*_c2_ around optimal doping. In this case one can write ${({H}_{{\rm{c}}2}^{^{\prime} }/{T}_{{\rm{c}}})}^{0.5}\propto {H}_{{\rm{c}}2}{\mathrm{(0)}}^{0.5}/{T}_{{\rm{c}}}\propto {v}_{{\rm{F}}1}^{-1}\propto {m}_{1}^{\ast }$. This explains the observed weak doping dependence of these quantities (Fig. 3). The obtained doping dependences of the (normalized reciprocal) *v*_F1_ and *v*_F2_ are shown in Fig. 3b. The 1/*v*_F1_ values are indeed smaller than 1/*v*_F2_ and show a weaker doping dependence. In contrast, 1/*v*_F2_ is strongly enhanced around optimal doping. The Ω_sf_ value affects the Fermi velocities quantitatively but their qualitative doping dependence is conserved. The corresponding normalized effective mass m*/*m*_b_ obtained from the de Haas-van Alphen (dHvA) experiments^7, 16^ (m_b_ is the quasiparticle mass taken from the band structure calculations) follows the same trend if the small shift of the QCP along the doping axis (Δ*x* = −0.05) due to the strain is taken into account (see Fig. 1b). The logarithmic divergence at *x*_c_ = 0.25 is an indication for the reduction of *v*_F2_ caused by the quantum fluctuations associated with a QCP of the SDW phase^8, 16^. A strong reduction of *v*_F2_ is observed also at *x* < 0.15 which roughly corresponds to the doping level where *T*_N_ > *T*_c_ (Fig. 1b, see also Tables S1 and S2 in the Supplementary material). This behavior may be associated with the reconstruction of the Fermi surface due to the presence of the coexisting SDW phase^15, 33, 34^.

Some of the multi-band heavy fermion superconductors show a similar behavior around the magnetic QCP as the BaFe_2_(As_1−*x*_P_*x*_)_2_ system. The measured enhancement of the effective mass depends also essentially on the experimental method^35^. Also, a seemingly conflicting behavior between the dHvA, ARPES and transport data was discussed for cuprate superconductors around optimal hole doping^36^. It was proposed that for the suggested nodal electron pocket induced by bidirectional charge order in high fields, the mass enhancement is very anisotropic around the small Fermi surface. It was argued that the corners of that pocket exhibit a large enhancement without any enhancement along the diagonal nodal direction. Such an angle-dependent mass enhancement is interpreted as a destruction of the Landau quasiparticles at ‘hot spots’ on the large Fermi surface at a proximate QCP. Moreover, another recent theoretical work questioned the paradigm of the universal nFL behavior at a QCP^37^. It was shown that at the nematic QCP the thermodynamics may remain of FL type, while, depending on the Fermi surface geometry, either the entire Fermi surface stays cold, or at most there are ‘hot spots’. Therefore, one may speculate that the complex behavior observed in FBS and in particular for BaFe_2_(As_1−*x*_P_*x*_)_2_ can be related to the superposition of *two distinct* QCPs associated with the SDW phase and the nematic order^38^. The evidence for two distinct QCPs was indeed reported for the Ba(Fe_1−*x*_Ni_*x*_)_2_As_2_ system^39^. Recently, a band-dependent mass enhancement toward the QCP was suggested from the high-field specific heat measurements of overdoped BaFe_2_(As_1−*x*_P_*x*_)_2_ single crystals^40^. Thus so far, the available experimental data emphasize the relevance of multi-band effects for a proper and complete understanding of the quantum criticality of BaFe_2_(As_1−*x*_P_*x*_)_2_ and related systems. Further experimental and theoretical investigations including possible strong coupling interactions since the suggested bosonic frequencies (spin fluctuations) exceed the superconducting critical temperature by a factor of three,only, retardation effects might be important, would be helpful to develop a microscopic scenario of the QCP for the title compound and other multi-band systems.

## Methods

### Samples

BaFe_2_(As_1−*x*_P_*x*_)_2_ single crystalline thin films with various P doping levels *x* were grown by MBE with a background pressure of the order of 10^−7^ Pa. All elements were supplied from solid sources charged in Knudsen cells. Pure elements were used as sources for Ba, Fe, and As. The P_2_ flux was supplied from a GaP decomposition source where Ga was removed by two trapping caps placed on the crucible. The details of the sample preparation are given in refs. 22 and 23. Some of the films on MgO (100) substrate were prepared by PLD with a KrF excimer laser (248 nm). In this case, we used polycrystalline BaFe_2_(As_1−*x*_P_*x*_)_2_ as the PLD target material. The preparation process took place in an ultra-high vacuum chamber with a similar base pressure of 10^−7^ Pa. Before the deposition, the substrate was heated to 850 °C. Then the BaFe_2_(As_1−*x*_P_*x*_)_2_ layer was grown with a laser repetition rate of 3 Hz. The layer thickness was adjusted via the pulse number at constant laser energy. To improve the sample’s homogeneity and thickness gradient, the substrate was rotated during the whole deposition process. Phase purity and crystalline quality of the films were examined by X-ray diffraction (XRD). The *c*-axis lattice parameters were calculated from the XRD data using the Nelson Riley function. It depends linearly on the P-doping (determined by electron probe micro-analysis (EPMA)) for the films grown on the same substrate^23^. In this work, we mainly investigated films prepared on MgO (100) substrate. At high doping levels, also several films on LaAlO_3_ (100) substrate have been used. The P-doping levels given in the paper have been determined using the *c*-axis lattice parameter values according to the data in ref. 23 as shown in the Supplementary material Fig. S1.

### Resistivity measurements

The temperature dependence of the electrical resistivity was measured by a four-contact method in a Quantum Design physical property measurement system (PPMS) in magnetic fields up to 14 T. Examples of the temperature dependence of the resistivity in zero and applied magnetic fields are shown in Supplementary material (Figs. S2–S7). The high-field measurements were performed in DC magnetic fields up to 35 T at the National High Magnetic Field Laboratory, Tallahassee, FL, USA. The high-field transport measurements in pulsed magnetic fields up to 67 T were performed at the Dresden High Magnetic Field Laboratory at HZDR and at the National High Magnetic Field Laboratory, Los Alamos, NM, USA. The superconducting transition temperature *T*_*c*_, as given in the paper, was determined using *T*_c,90_ as shown in the Supplementary material (Figs. S6 and S7). Other criteria, such as 50% of the normal state resistance, yield qualitatively the same temperature dependence of *H*_c2_. The SDW transition temperature *T*_N_ was defined as the peak position of the temperature derivative of the resistivity curves in analogy to the procedure applied for bulk single crystals^41^, see Supplementary material Fig. S2.

The measurements were performed in magnetic fields applied along the crystallographic *c*-axis of the films, which coincides with the normal direction of the films surface. Therefore, the *H*_c2_ data presented in the paper depend on the in-plane coherence length ${\xi }_{{\rm{ab}}}$ only, which is unaffected by the film thickness *D*_film_ ~ 100 nm. Additionally, ${\xi }_{c} > d/2$ is satisfied for all doping levels, where *d* is the spacing between the neighboring FeAs layers. The estimates given in the Supplementary material indicate that the fluctuation effects close to *T*_c_ can be neglected in our case. We assume that the transition width is related to small inhomogeneities in the P distribution and to a difference between *H*_c2_(*T*) and *H*_irr_(*T*), where *H*_irr_ is the irreversibility field. In particular, *H*_irr_(*T*) is noticeably affected by flux pinning at low temperatures and high magnetic fields. Thus, our consideration of BaFe_2_(As_1−*x*_P_*x*_)_2_ thin films as 3D superconductors and the neglect of 2D corrections and fluctuation effects are indeed justified.

## Electronic supplementary material

SUPPLEMENTARY MATERIAL: SELECTIVE MASS ENHANCEMENT CLOSE TO THE QUANTUM CRITICAL POINT IN BaFe2(As1-xPx)2

## References

[1] Scalapino DJ (2012). A common thread: The pairing interaction for unconventional superconductors. Rev. Mod. Phys..

[2] Hirschfeld MM, Korshunov PJ, Mazin II (2011). Gap symmetry and structure of Fe-based superconductors. Rep. Prog. Phys..

[3] Gabovich AI, Voitenko AM, Ausloos M (2002). Charge- and spin-density waves in existing superconductors: competition between cooper pairing and peierls or excitonic instabilities. Physics Reports.

[4] Gegenwart Q, Si P, Steglich F (2008). Quantum criticality in heavy-fermion metals. Nat. Phys..

[5] Bauer E (2010). Pressure-induced superconducting state and effective mass enhancement near the antiferromagnetic quantum critical point of CePt_2_In_7_. Phys. Rev. B.

[6] Johnston DC (2010). The puzzle of high temperature superconductivity in layered iron pnictides and chalcogenides. Advances in Physics.

[7] Shishido H (2010). Evolution of the fermi surface of BaFe_2_(As_1−*x*_P_*x*_)_2_ on entering the superconducting dome. Phys. Rev. Lett..

[8] Analytis JG (2014). Transport near a quantum critical point in BaFe_2_(As_1−*x*_P_*x*_)_2_. Nat. Phys.

[9] Hayes, I. M. *et al*. Scaling between magnetic field and temperature in the high-temperature superconductor BaFe_2_(As_1−*x*_P_*x*_)_2_. *Nat*. *Phys*. **12**, 916–919, doi:10.1038/nphys3773 (2016).

[10] Nakai Y (2010). Unconventional superconductivity and antiferromagnetic quantum critical behavior in the isovalent-doped BaFe_2_(As_1−*x*_P_*x*_)_2_. Phys. Rev. Lett..

[11] Iye T (2012). Gradual suppression of antiferromagnetism in BaFe_2_(As_1−*x*_P_*x*_)_2_: Zero-temperature evidence for a quantum critical point. Phys. Rev. B.

[12] Nakai Y (2013). Normal-state spin dynamics in the iron-pnictide superconductors BaFe_2_(As_1−*x*_P_*x*_)_2_ and Ba(FeCo_1−*x*_)_2_As_2_ probed with nmr measurements. Phys. Rev. B.

[13] Abrahams E, Si Q (2011). Quantum criticality in the iron pnictides and chalcogenides. J. Phys.: Condens. Matter.

[14] Shibauchi A, Carrington T, Matsuda Y (2014). A quantum critical point lying beneath the superconducting dome in iron pnictides. Annu. Rev. Condens. Matter Phys..

[15] Fink J (2015). Non-fermi-liquid scattering rates and anomalous band dispersion in ferropnictides. Phys. Rev. B.

[16] Walmsley P (2013). Quasiparticle mass enhancement close to the quantum critical point in BaFe_2_(As_1−*x*_P_*x*_)_2_. Phys. Rev. Lett..

[17] Hashimoto K (2012). A sharp peak of the zero-temperature penetration depth at optimal composition in BaFe_2_(As_1−*x*_P_*x*_)_2_. Science.

[18] Lamhot Y (2015). Local characterization of superconductivity in BaFe_2_(As_1−*x*_P_*x*_)_2_. Phys. Rev. B.

[19] Levchenko A (2013). Enhancement of the london penetration depth in pnictides at the onset of spin-density-wave order under superconducting dome. Phys. Rev. Lett..

[20] Nomoto T, Ikeda H (2013). Effect of magnetic criticality and fermi-surface topology on the magnetic penetration depth. Phys. Rev. Lett..

[21] Putzke C (2014). Anomalous critical fields in quantum critical superconductors. Nat. Commun..

[22] Kurth F (2015). Unusually high critical current of clean P-doped BaFe_2_As_2_ single crystalline thin films. Appl. Phys. Lett..

[23] Kawaguchi T (2014). The strain effect on the superconducting properties of BaFe_2_(As,P)_2_ thin films grown by molecular beam epitaxy. Supercond. Sci. Technol..

[24] Sato H (2014). High critical-current density with less anisotropy in BaFe_2_(As,P)_2_ epitaxial thin films: Effect of intentionally grown c-axis vortex-pinning centers. Appl. Phys. Lett..

[25] Kasahara S (2010). Evolution from non-fermi- to fermi-liquid transport via isovalent doping in BaFe_2_(As_1−*x*_P_*x*_)_2_ superconductors. Phys. Rev. B.

[26] Iida K (2009). Strong *T*_*c*_ dependence for strained, epitaxial Ba(Fe_1−*x*_Co_*x*_)_2_As_2_ thin films. Appl. Phys. Lett..

[27] Engelmann J (2013). Strain induced superconductivity in the parent compound BaFe_2_As_2_. Nat. Commun..

[28] Iida K (2016). Hall-plot of the phase diagram for Ba(Fe_1−*x*_Co_*x*_)_2_As_2_. Sci. Rep..

[29] Werthamer E, Helfand NR, Hohenberg PC (1966). Temperature and purity dependence of the superconducting critical field, *H*_c2_. III. electron spin and spin-orbit effects. Phys. Rev..

[30] Gurevich A (2010). Upper critical field and the fulde-ferrel-larkin-ovchinnikov transition in multiband superconductors. Phys. Rev. B.

[31] Gurevich A (2011). Iron-based superconductors at high magnetic fields. Rep. Prog. Phys..

[32] C. H. Lee, *et al*. Universality of the Dispersive Spin-Resonance Mode in Superconducting. *Phys. Rev. Lett*. **111**, 167002 (2013). 10.1103/PhysRevLett.111.167002 24182293

[33] Yi M (2012). Electronic reconstruction through the structural and magnetic transitions in detwinned nafeas. New J. of Phys..

[34] Nakashima Y (2013). Fermi-surface reconstruction involving two van hove singularities across the antiferromagnetic transition in BaFe_2_As_2_. Solid State Commun..

[35] Knebel G (2008). The quantum critical point in cerhin_5_: A resistivity study. J. Phys. Soc. Jpn..

[36] Senthil, T. On the mass enhancement near optimal doping in high magnetic fields in the cuprates. *arXiv*:*1410*.*2096* (2014).

[37] Paul, I. & Garst, M. Lattice effects on nematic quantum criticality in metals. *Phys. Rev. Lett.* **118**, 227601 (2017). 10.1103/PhysRevLett.118.227601 28621984

[38] Chowdhury D (2015). Phase transition beneath the superconducting dome in BaFe_2_(As_1−*x*_P_*x*_)_2_. Phys. Rev. B.

[39] Zhou R (2013). Quantum criticality in electron-doped BaFe_2−*x*_Ni_*x*_As_2_. Nature Comm..

[40] Moir, C. M. *et al*. Mass enhancement in multiple bands approaching optimal doping in a high-temperature superconductor. *arXiv*:*1608*.*07510* (2016).

[41] Pratt DK (2009). Coexistence of competing antiferromagnetic and superconducting phases in the underdoped Ba(Fe_0.953_Co_0.047_)_2_As_2_ compound using x-ray and neutron scattering techniques. Phys. Rev. Lett..

