# Supplementary material for: Selective mass enhancement close to the quantum critical point in BaFe2(As1−xPx)2
Source: Sci Rep. 2017 Jul 4;7:4589. doi: 10.1038/s41598-017-04724-3 (PMC5496881; doi:10.1038/s41598-017-04724-3)
Supplement: Supplementary file 1 — SUPPLEMENTARY MATERIAL: SELECTIVE MASS ENHANCEMENT CLOSE TO THE QUANTUM CRITICAL POINT IN BaFe2(As1-xPx)2 [file 41598_2017_4724_MOESM1_ESM.pdf]

SUPPLEMENTARY MATERIAL: SELECTIVE MASS ENHANCEMENT CLOSE TO THE QUANTUM CRITICAL POINT IN $BaFe_2(As_{1-x}P_x)_2$

V. Grinenko[1,2,3,*], K. Iida[2,3], F. Kurth[1,2], D. V. Efremov[2], S.-L. Drechsler[2], I. Cherniavskii[4], I. Morozov[2,4], J. Hänisch[2,5], T. Förster[6], C. Tarantini[7], J. Jaroszynski[7], B. Maiorov[8], M. Jaime[8], A. Yamamoto[9], I. Nakamura[3], R. Fujimoto[3], T. Hatano[3], H. Ikuta[3], AND R. Hühne[2]

1. Institute for Solid State Physics, TU Dresden, 01069 Dresden, Germany

2. IFW Dresden, Helmholtzstrasse 20, 01069 Dresden, Germany

3. Department of Crystalline Materials Science, Graduate School of Engineering, Nagoya University, Furo-cho, Chikusa-ku, Nagoya 464-8603, Japan

4. Lomonosov Moscow State University, GSP-1, Leninskie Gory, Moscow, 119991, Russian Federation

5. Karlsruhe Institute of Technology, Institute for Technical Physics, Hermann-von-Helmholtz-Platz 1, 76344 Eggenstein-Leopoldshafen, Germany

6. Hochfeld-Magnetlabor Dresden (HLD-EMFL), Helmholtz-Zentrum Dresden-Rossendorf, 01314 Dresden, Germany

7. NHMFL, Florida State University, Tallahassee, FL 32310, USA

8. MPA-CMMS, Los Alamos National Laboratory, Los Alamos, NM, 87545, USA

9. Department of Applied Physics, Tokyo University of Agriculture and Technology 2-24-16 Nakacho, Koganei, Tokyo 184-8588, Japan

In the present Supplementary material we show the doping dependence of the c-axis lattice parameter for $BaFe_2(As_{1-x}P_x)_2$ films grown on MgO and $LaAlO_3$ (LAO) substrates, the temperature dependences of the electrical resistance in magnetic fields, the criteria used for the determination of the SDW transition temperature T_N and the superconducting critical temperature T_c in static magnetic fields as well as the upper critical field H_{c2} in pulsed magnetic fields. We estimated the fluctuation effect on the superconducting transition width and that on the evaluated values of the slope of the upper critical field. Finally, we provide tables with a list of the fitting parameters described in the main text.

Composition of the films. The P doping level of the thin films given in the main text were calculated using the relation between the c-axis lattice parameter and the P doping obtained in previous studies (Fig. S1).[S1]

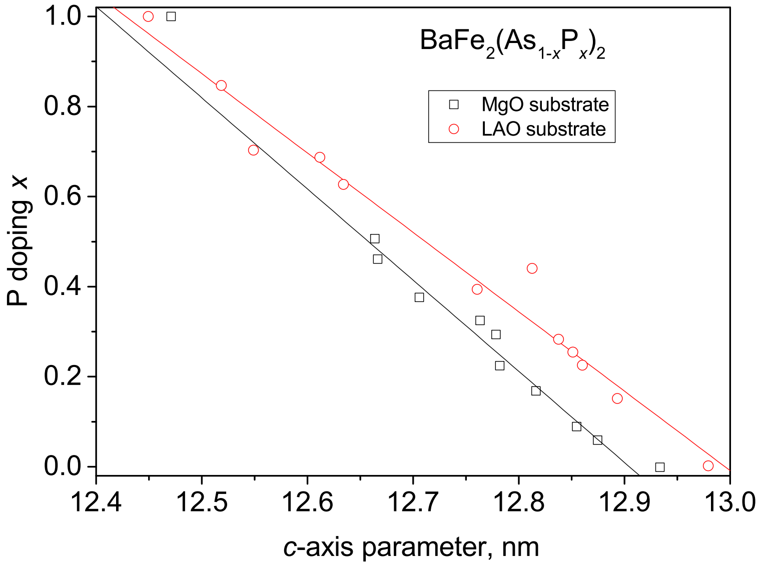

FIGURE S1. (a) Relation between the c-axis lattice parameter and the P doping level x of $BaFe_2(As_{1-x}P_x)_2$ films grown on MgO and $LaAlO_3$ (LAO) substrates. The data are taken from previous studies.[S1]

Evaluation of $T_{\rm N}$ and $T_{\rm c}$. The spin density wave transition temperatures $T_{\rm N}$ of the $BaFe_2(As_{1-x}P_x)_2$ films were defined by a standard procedure developed for single crystals (Fig. S2).[S2]

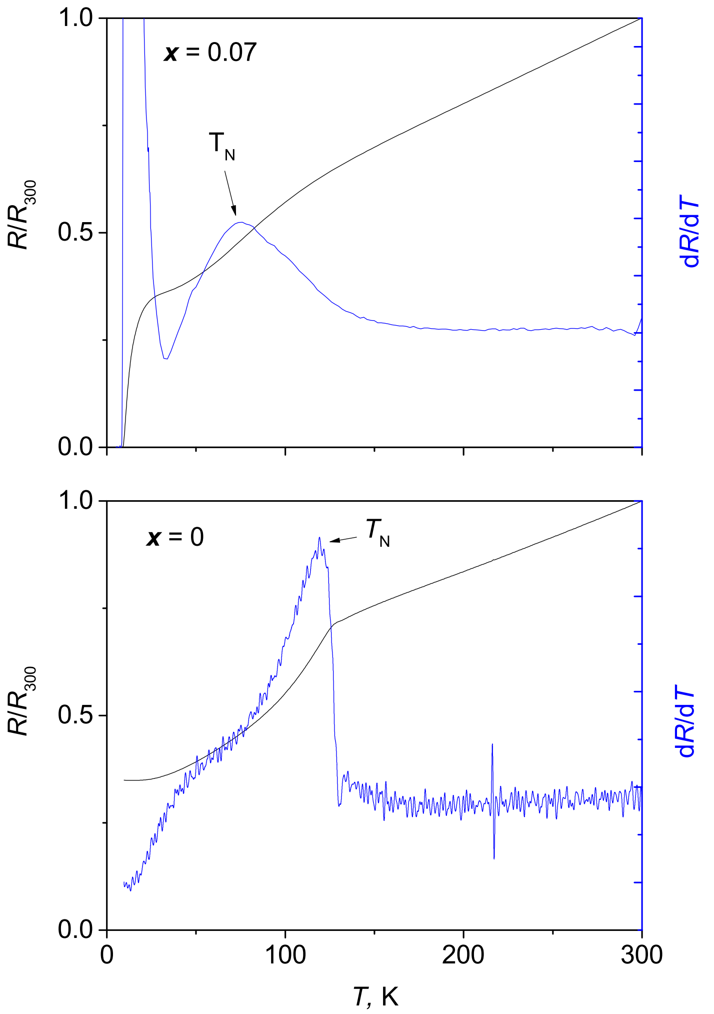

FIGURE S2. An example of the temperature dependence of the resistance of $BaFe_2(As_{1-x}P_x)_2$ films with SDW transition at low temperatures (left) and its derivative (right). The peak position of the derivative is assigned as $T_{\rm N}$. The criterion is based on the comparison between the neutron scattering and transport data for the $BaFe_2As_2$ system.[S2]

The temperature dependencies of the upper critical fields H_{c2} given in the main text were obtained from resistivity measurements in static and pulsed magnetic fields (Figs. S3, S4, and S5).

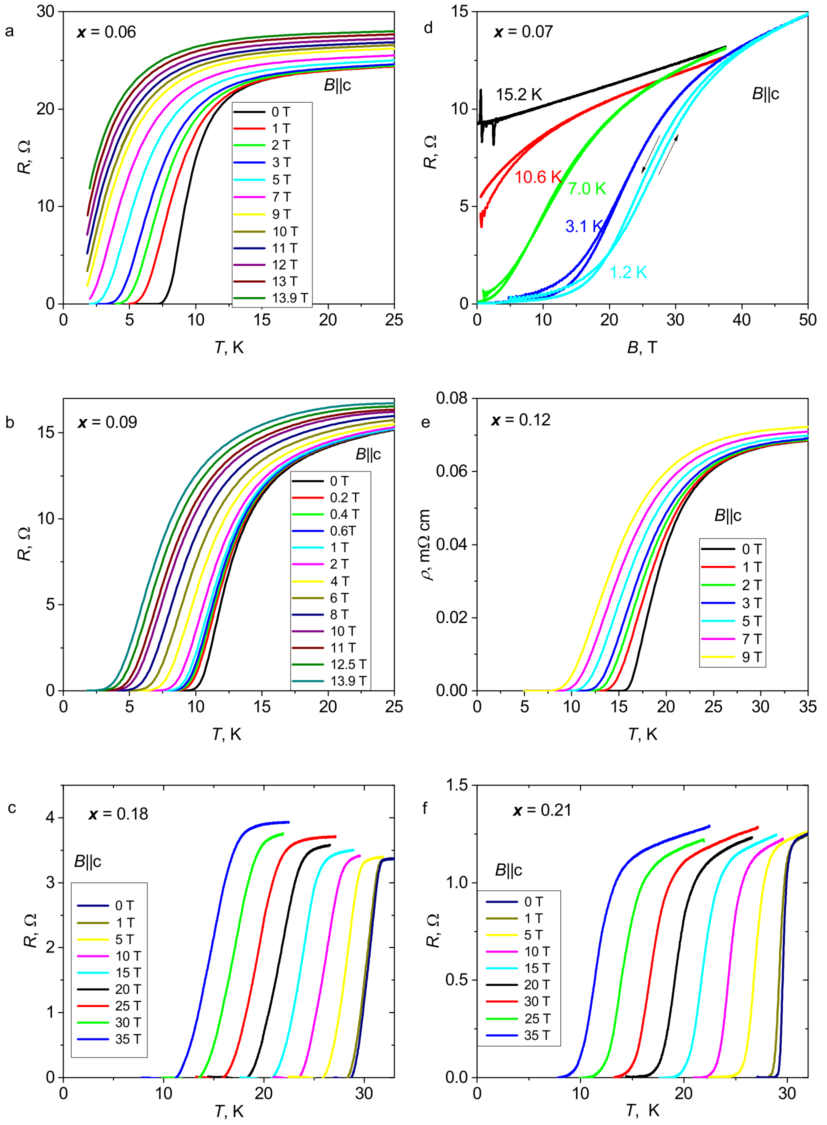

FIGURE S3. Temperature dependences of the resistance (resistivity if available) of $BaFe_2(As_{1-x}P_x)_2$ films prepared by MBE in static magnetic fields and field dependences of the resistivity in pulsed magnetic fields. The doping levels, fields strength and temperatures are given in the figures too.

We present two examples of the H_{c2} temperature dependences defined using different criteria $T_{c,on}$, $T_{c,90}$, and $T_{c,50}$ as shown in Figs. S6a and S7. In all cases, the different

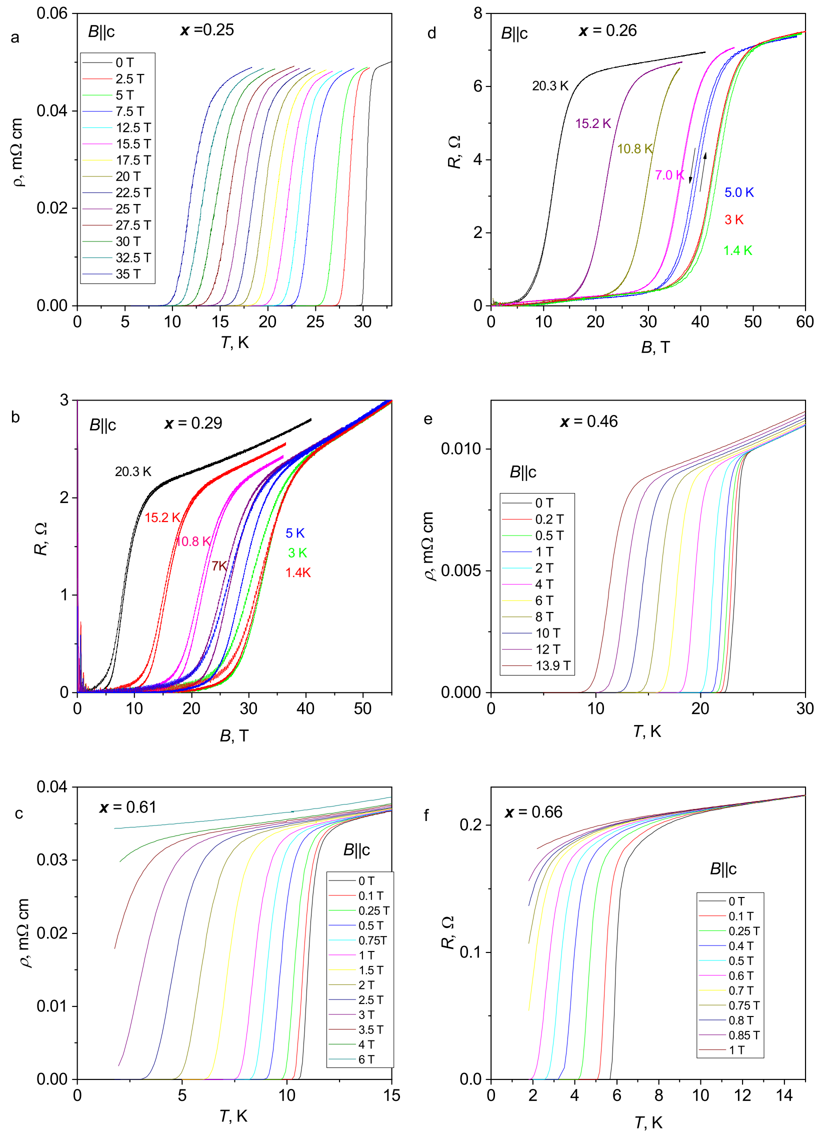

FIGURE S4. Temperature dependences of the resistance (resistivity if available) of $BaFe_2(As_{1-x}P_x)_2$ films prepared by MBE in static magnetic fields and field dependences of the resistivity in pulsed magnetic fields. The doping levels, fields strength and temperatures are given in the figures too.

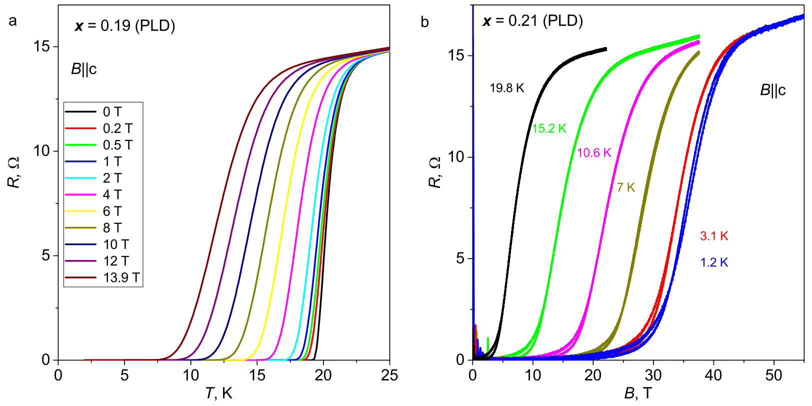

FIGURE S5. Temperature dependences of the resistance of $BaFe_2(As_{1-x}P_x)_2$ films prepared by PLD in static magnetic fields and field dependences of the resistivity in pulsed magnetic fields. The doping levels, fields strength and temperatures are given in the figures too.

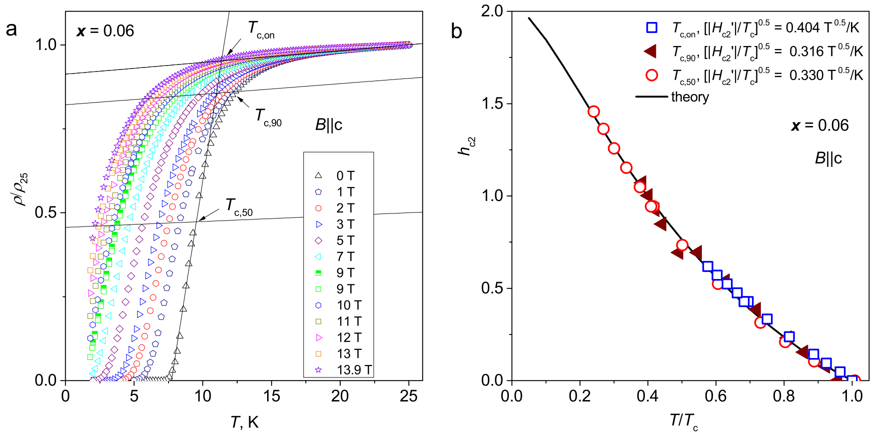

FIGURE S6. (a) Temperature dependences of the resistance of a $BaFe_2(As_{0.94}P_{0.06})_2$ film prepared by MBE in static magnetic fields. $T_{c,on}$, $T_{c,90}$, and $T_{c,50}$ denote different criteria used to plot the $h_{c2} = \frac{H_{c2}}{-H'_{c2}T_c}$ values versus reduced temperature T/T_c as shown in panel (b).

criteria result in a quantitative but not a qualitative change of the H_{c2} dependences (Figs. S6b, and S7b). To exclude possible effects of the irreversibility field H_{irr} on the transition width, in the main text we used $T_{c,90}$ to plot H_{c2} (for discussion see below).

Two-band model for H_{c2}. The doping evolution of the temperature dependencies of H_{c2} was described by the two-band model for a clean superconductor as proposed by Gurevich [S9, S10]. Its expression for $B \parallel c$ is given by

$$a_1 G_1 + a_2 G_2 + G_1 G_2 = 0, \tag{S1}$$

where

$$G_1 = \ln t + 2e^{q^2}\mathrm{Re}\sum_{n=0}^{\infty}\int_0^{\infty} e^{-u^2}\left[\frac{u}{n+1/2} - \frac{t}{\sqrt{b}}\tan^{-1}\left(\frac{u\sqrt{b}}{t(n+1/2)+\mathrm{i}\alpha b}\right)\right]du,$$

G_2 is obtained by substituting $\sqrt{b} \to \sqrt{\eta b}$ and $q \to q\sqrt{s}$ in G_1,

$a_1 = (\lambda_0 + \lambda_-)/2\omega, a_2 = (\lambda_0 - \lambda_-)/2\omega,$

$\lambda_- = \lambda_{11} - \lambda_{22}, \lambda_0 = (\lambda_-^2 + 4\lambda_{12}\lambda_{21})^{1/2}, \omega = \lambda_{11}\lambda_{22} - \lambda_{12}\lambda_{21},$

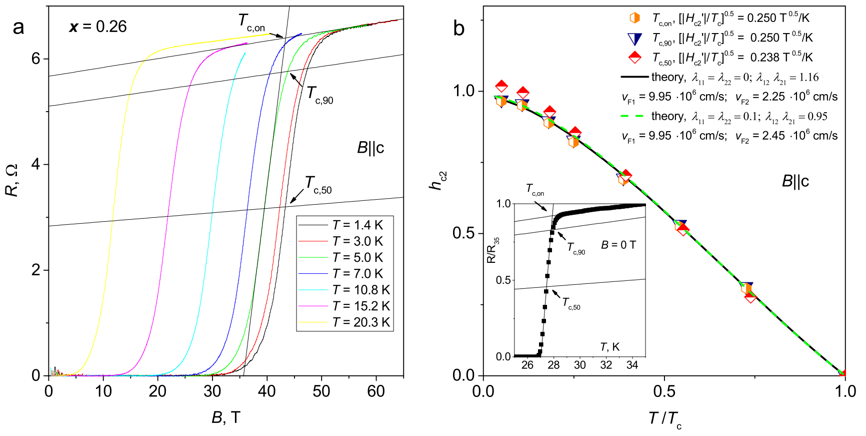

FIGURE S7. (a) Field dependences of the resistance of a $BaFe_2(As_{0.74}P_{0.26})_2$ film prepared by MBE (pulsed field measurements). $T_{c,on}$, $T_{c,90}$, and $T_{c,50}$ denote different criteria used to plot the $h_{c2} = \frac{H_{c2}}{-H'_{c2}T_c}$ values versus reduced temperature T/T_c as shown in panel (b). The temperature dependence of the normalized resistance R/R_{35} is shown in the inset of panel (b). The fitting curves correspond to two different cases with zero intraband coupling constants - solid line and non-zero intraband coupling constants - dased line, where $\Omega_{sf} = 62$ K.

and $b = \frac{\hbar^2 v_{\rm F1}^2 H}{8\pi\phi_0 k_{\rm B}^2 T_{\rm c}^2}$, $q^2 = \frac{Q_z^2 \varepsilon_1 \phi_0}{2\pi H}$, $\alpha = \frac{4\mu\phi_0 T_{\rm c}}{\hbar^2 v_{\rm F1}^2}$,

where $t = T/T_{\rm c}$, $\eta = (v_{\rm F2}/v_{\rm F1})^2$, $s = \varepsilon_2/\varepsilon_1$, $v_{{\rm F}i}$ is the in-plane Fermi velocity in band $i = 1, 2$, and $\varepsilon_i = m_i^{ab}/m_i^c$ is the mass anisotropy ratio, ϕ_0 is the flux quantum, μ is the magnetic moment of a quasiparticle, λ_{ii} intraband and λ_{ij}, ($j = 1, 2$, and $i \neq j$) interband pairing constants, and $\alpha \approx \alpha_{\rm M}/1.8$, where the Maki parameter $\alpha_{\rm M} = 2^{1/2} H_{\rm c2}^{\rm orb}/H_{\rm p}$ quantifies the strength of the paramagnetic pair breaking. The Fulde-Ferrel-Larkin-Ovchinnikov (FFLO) wave vector **Q** is determined by the condition that $H_{\rm c2}$ is at maximum, where Q_Z is its projection onto c-axis.

Effect of inhomogeneities and fluctuations on the superconducting transition width. The underdoped films have a relatively broad transition to the superconducting state $\Delta T_{\rm c}$ (see Figs. S3 and S6a). This broadening of the transition can be explained by a small inhomogeneity of the P doping within $\sim 1\%$ together with a strong doping dependency of $T_{\rm c}$ in the coexistence state between SDW and superconductivity and possible additional broadening in an applied magnetic field due to the irreversibility field $H_{\rm irr}$. For type-II superconductors in applied magnetic fields the temperature where $R = 0$ depends on the strength of vortex pinning, i.e. the condition where the critical current $I_{\rm c} = 0$ (for sufficiently small measurement currents) and not by $H_{\rm c2}$ which is related to the condition when the normal vortex cores overlap. Therefore, $R = 0$ corresponds to the irreversibility line $H_{\rm irr}$ rather than to $H_{\rm c2}$. In general, $H_{\rm irr}$ can differ considerably from $H_{\rm c2}$.[S3, S4] Therefore, we avoided to employ this $R = 0$ criterion. We note that the high-field data presented in Ref. [S5] reflect the measurements of $H_{\rm irr}$, which actually differs from $H_{\rm c2}$.

TABLE S1. The parameters obtained from the fit of H_{c2} temperature dependences shown in Fig. 2 main text. $\Omega_{sf} = 100$ K. The crystallographic c-axis length is given in nm, the Fermi velocities are given in 10^6 cm s^{-1} and the transition temperature is in K.

Substrate/technique	c - axis, Å	P - doping	v_{F1}	v_{F2}	$[\lambda_{12}\lambda_{21}]^{0.5}$	T_c
MgO/MBE	12.877	0.056	8.4	0.65	0.43	11.4
MgO/MBE	12.870	0.070	6.4	1.60	0.48	14.0
MgO/MBE	12.815	0.182	8.5	4.07	0.78	31.7
MgO/MBE	12.800	0.212	9.3	3.90	0.75	30.3
MgO/MBE	12.782	0.248	10.1	3.05	0.76	30.7
MgO/MBE	12.774	0.263	9.8	3.05	0.73	27.9
MgO/MBE	12.761	0.291	10.6	3.50	0.69	26.9
MgO/PLD	12.811	0.190	6.0	5.80	0.70	21.6
MgO/PLD	12.800	0.212	9.3	3.08	0.75	24.9
LAO/MBE	12.736	0.457	11.1	4.15	0.64	23.7
LAO/MBE	12.649	0.610	11.4	6.46	0.44	11.4
LAO/MBE	12.619	0.663	11.3	7.90	0.34	6.3

TABLE S2. The parameters obtained from the fit of H_{c2} temperature dependences shown in Fig. 2 main text. $\Omega_{sf} = 62$ K. The crystallographic c-axis length is given in nm, the Fermi velocities are given in 10^6 cm s^{-1} and the transition temperature is in K.

Substrate/technique	c - axis, Å	P - doping	v_{F1}	v_{F2}	$[\lambda_{12}\lambda_{21}]^{0.5}$	T_c
MgO/MBE	12.877	0.056	8.20	0.61	0.55	11.4
MgO/MBE	12.870	0.070	6.50	1.37	0.62	14.0
MgO/MBE	12.815	0.182	8.50	3.80	1.25	31.7
MgO/MBE	12.800	0.212	9.70	3.00	1.18	30.3
MgO/MBE	12.782	0.248	10.20	2.20	1.20	30.7
MgO/MBE	12.774	0.263	9.95	2.25	1.08	27.9
MgO/MBE	12.761	0.291	11.05	2.45	1.03	26.9
MgO/PLD	12.811	0.190	6.00	5.80	1.20	21.6
MgO/PLD	12.800	0.212	9.60	2.60	0.96	24.9
LAO/MBE	12.736	0.457	11.30	3.55	0.91	23.7
LAO/MBE	12.649	0.610	11.60	6.23	0.55	11.4
LAO/MBE	12.619	0.663	11.40	7.80	0.41	6.3

The effect of superconducting fluctuations plays a secondary role for the ΔT_c values of our thin films. For example, the films with the highest $T_c \sim 30$ K have a transition

width of about 1 K, only. Note that, even these transitions width are not yet dominated by fluctuations. Quantitatively, one can estimate the temperature range where fluctuations play a role using the Ginzburg parameter defined in 3D (see also Methods section in the main text) as $\Delta T_{\rm c}/T_{\rm c} = Gi \approx 80(T_{\rm c}/E_{\rm F})^4 \sim 10^{-4} - 10^{-5}$.[S6] Taking $T_{\rm c} = 30$ K and the largest $E_{\rm F} \sim 100$ meV in our system according to ARPES data.[S7] Alternatively, the Gi parameter can be estimated directly from the superconducting parameters: $\Delta T_{\rm c}/T_{\rm c} = Gi = (\Gamma k_{\rm B}T_{\rm c}/H_{\rm cm}(0)^2\xi_0^3)^2/2$.[S3, S8] Taking the experimental values for $T_{\rm c} = 30$ K, $H_{\rm c2}$ = 470 kG, $H_{\rm c1}$ = 600 G (field along crystallographic c-axis),[S5] $\lambda \approx 3\cdot 10^{-5}$ cm, $\xi_{\rm c} = [\Phi_0/2\pi H_{\rm c2}]^{0.5} \approx 3\cdot 10^{-7}$ cm and the anisotropy $\Gamma \sim 2$ - 4, one arrives at the same estimates. However, close to $T_{\rm c}$ the c-axis coherence length $\xi_{\rm c}(T)$ diverges as $(1 - T/T_{\rm c})^{-0.5}$. Therefore, in close vicinity to $T_{\rm c}$, where $\xi_{\rm c}(T) > D_{\rm film}$, one can consider the films as 2D superconductors, where $D_{\rm film} \approx 100$ nm is the films thickness. For optimally doped films with high $H_{\rm c2}$ values one can estimate that $\xi_{\rm c}(T) \sim D_{\rm film}$ holds only for a very narrow temperature range of $\Delta T/T_{\rm c} \approx 0.001$. On the other hand, this range is about 0.5 K for overdoped films with low $T_{\rm c} \sim 10$ K and small $H_{\rm c2} \sim 10$ kG. However, due to the low $T_{\rm c}$, the fluctuation effect is rather weak $\Delta T_{\rm c}/T_{\rm c} = Gi \approx (T_{\rm c}/E_{\rm F}) \sim 10^{-3}$ even in 2D case.

Finally we list parameters obtained from the fits of $H_{\rm c2}$ temperature dependences shown in the main text (Tabs. S1, and S2), for the case of a zero intraband coupling $\lambda_{11} = \lambda_{22} = 0$.

REFERENCES

[S1] T. Kawaguchi, A. Sakagami, Y. Mori, M. Tabuchi, T. Ujihara, Y. Takeda, and H. Ikuta, The strain effect on the superconducting properties of $BaFe_2(As,P)_2$ thin films grown by molecular beam epitaxy, Supercond. Sci. Technol. **27**, 065005 (2014).

[S2] D. K. Pratt, W. Tian, A. Kreyssig, J. L. Zarestky, S. Nandi, N. Ni, S. L. Budko, P. C. Canfield, A. I. Goldman, and R. J. McQueeney, Coexistence of Competing Antiferromagnetic and Superconducting

Phases in the Underdoped Ba($Fe_{0.953}Co_{0.047}$)$_2$As$_2$ Compound Using X-ray and Neutron Scattering Techniques, Phys. Rev. Lett. **103**, 087001 (2009).

[S3] G. Blatter, M. V. Feigel'man, V. B. Geshkenbein, A. I. Larkin, and V. M. Vinokur, Vortices in high-temperature superconductors, Rev. Mod. Phys. **66**, 1125 (1994).

[S4] R. Prozorov, N. Ni, M. A. Tanatar, V. G. Kogan, R. T. Gordon, C. Martin, E. C. Blomberg, P. Prommapan, J. Q. Yan, S. L. Budko, and P. C. Canfield, Vortex phase diagram of Ba($Fe_{0.93}Co_{0.07}$)$_2$As$_2$ single crystals, Phys. Rev. B **78**, 224506 (2008).

[S5] C. Putzke, P. Walmsley, J. D. Fletcher, L. Malone, D. Vignolles, C. Proust, S. Badoux, P. See, H. E. Beere, D. A. Ritchie, S. Kasahara, Y. Mizukami, T. Shibauchi, Y. Matsuda, A. Carrington, Anomalous critical fields in quantum critical superconductors, Nat. Commun. **5**, 5679 (2014).

[S6] A. Larkin and A. Varlamov, Fluctuation Phenomena in Superconductors, arXiv:cond-mat/0109177.

[S7] T. Yoshida, I. Nishi, S. Ideta, A. Fujimori, M. Kubota, K. Ono, S. Kasahara, T. Shibauchi, T. Terashima, Y. Matsuda, H. Ikeda, and R. Arita, Two-Dimensional and Three-Dimensional Fermi Surfaces of Superconducting BaFe$_2$(As$_{1-x}$P$_x$)$_2$ and Their Nesting Properties Revealed by Angle-Resolved Photoemission Spectroscopy, Phys. Rev. Lett. **106**, 117001 (2011).

[S8] C. Chaparro, L. Fang, H. Claus, A. Rydh, G. W. Crabtree, V. Stanev, W. K. Kwok, and U. Welp, Doping dependence of the specific heat of single-crystal BaFe$_2$(As$_{1-x}$P$_x$)$_2$, Phys. Rev. B **85**, 184525 (2012).

[S9] A. Gurevich, Upper critical field and the Fulde-Ferrel-Larkin-Ovchinnikov transition in multiband superconductors, Phys. Rev. B **82**, 184504 (2010).

[S10] A. Gurevich, Iron-based superconductors at high magnetic fields, Rep. Prog. Phys. **74**, 124501 (2011).
